# Accuracy of SCORTEN in predicting mortality in toxic epidermal necrolysis

**DOI:** 10.1186/s12911-022-02013-2

**Published:** 2022-10-19

**Authors:** Jerzy Strużyna, Agnieszka Surowiecka, Tomasz Korzeniowski, Patrycja Korulczyk, Lukasz Drozd, Aldona Stachura, Kamil Torres, Andrzej Krajewski

**Affiliations:** 1 East Center of Burns Treatment and Reconstructive Surgery, Medical University of Lublin, Łęczna, Poland; 2 Department of Plastic Surgery, Reconstructive Surgery and Burn Treatment, Medical University of Lublin, Łęczna, Poland; 3 Chair and Department of Didactics and Medical Simulation, Medical University of Lublin, Łęczna, Poland; 4 West Pomeranian Burns and Plastic Surgery Center, Gryfice, Poland

**Keywords:** Toxic epidermal necrolysis, Burn, Wound, Plasmapheresis, IVIGs

## Abstract

**Background:**

Toxic epidermal necrolysis (TEN) patients require multi-directional and multi-disciplinary treatment. In most cases, they are hospitalised at intensive care units and require multi-directional, burn-complication preventive care. Choosing the most appropriate treatment option might be troublesome even when predicting scores are used. SCORTEN is the most renowned prognostic score for TEN patients, however, there are some data indicating that the accuracy of this test may be limited. The credibility of not just the predicted mortality risk, but also componential laboratory results and clinical features subject to debate. The aim of this study was to evaluate the efficacy and credibility of SCORTEN in clinical practice, on proprietary material.

**Methods:**

A retrospective analysis of 35 patients with diagnosed in histopathology TEN was performed. The inclusion criteria were as follows: day of submission before 5th day from the onset of the symptoms, full protocol of plasmaphereses and IVIGs according to our scheme. Our protocol includes cycle of plasmapheresis with frozen fresh plasma twice daily for the first 2 days following admission, and once daily for the subsequent 5 to 7 days. IVIGs were administered after the first two sessions of plasmapheresis, for 4 to 7 days. The dosage was calculated according to body weight, at 0.4 to 0.5 g/kg per dose.

**Results:**

The sensitivity of SCORTEN for the analysed cohort was 100%, with a specificity of 24%. The estimated death was 41,9%, while the actual death rates were 12,5%. Our protocol improved the survival, OR = 26,57, RR = 6,34, p = 0,022. Decrease in mortality was caused by a combined treatment protocol we use- plasmaphereses with IVIGs. No independent risk factor was significant in death evaluation.

**Conclusion:**

Our data suggest that the scoring system for predicting death among TEN patients are reliable when they are high. New prognostic factors should be found to improve the evaluation of patients with low SCORTEN.

## Introduction

Toxic epidermal necrolysis (TEN) is a blistering disorder most often caused by a severe adverse reaction to drugs [1–3]. The appearance of blisters and skin erosion is preceded by prodromal influenza-like symptoms [4]. Toxic epidermal necrolysis is distinguished from Stevens-Johnson Syndrome (SJS) by a larger extent of the lesions, covering over 30% of the total body surface area (TBSA) in TEN, and higher mortality. Mechanisms leading to epidermal destruction and keratinocyte apoptosis in TEN [2, 14] are associated with the activation of cytotoxic T lymphocytes and a circulating pro-inflammatory cytokine [2]. Patients diagnosed with TEN require multi-directional and multi-disciplinary treatment. In most cases, they are hospitalised at intensive care units (ICUs) and require multi-directional preventive care for complications. According to the available data, the mean mortality rate among TEN patients treated in ICUs is currently approximately 50% [2, 5–7]. One of the severe complications in TEN patients is multi-organ failure (MOF) secondary to infection caused most often by *Staphylococcus aureus* or *Pseudomonas* species [8]. Hsu et al. reported that in a group of SJS/TEN patients the most common secondary diagnosis was MRSA, Pseudomonas aeruginosa, E.coli or Gram- negative septicemia [9]. Chronic preexisting conditions worsened the outcome [9].

The decrease in mortality that has been observed recently might be due to better wound care and sepsis control [10]. There are many factors that increase mortality in TEN. There are some independent factors described in SCORTEN, or pre-existing renal failure and haemodialysis mentioned in ABCD-10 score known for their crucial impact on patients’ survival in certain populations [11]. Several prognostic scores are used to predict mortality [12, 13]. Acute Physiology and Chronic Health Evaluation (APACHE II) [14], Charlson Comorbidity Index (CCI) [15] or Sequential Organ Failure Assessment (SOFA) [16] are the most popular scales used to estimate mortality in ICU patients. The most renowned of such scores was introduced by Bastuji-Garin et al. in 2000. SCORTEN, or the Severity-of-Illness Score for Toxic Epidermal Necrolysis, is a model consisting of seven individual risk factors for death. It includes patient age over 40, malignancies, tachycardia, initial extent of skin lesions, serum urea, as well as serum glucose and bicarbonate levels [17]. Cutting points were proposed for the individual mortality risk factor and a deviation in each is a point in the score. Up to 1 point refers to 3% mortality risk, while 5 points- 85% and > 6 points- 95%. Some authors use SCORTEN not only to predict mortality, but also to evaluate the efficacy of immunomodulatory treatment [18, 19] or supportive therapy evaluation. However, there is evidence suggesting that SCORTEN tends to overestimate mortality risk in certain populations [20].

The aim of our study was to verify the accuracy of SCORTEN in our cohort, as well as to verify the significance of individual SCORTEN parameters for death prediction.

## Materials and methods

The study is a retrospective evaluation of clinical data of TEN patients treated at the East Centre of Burns Treatment and Reconstructive Surgery, Łęczna, Poland, between 2010 and 2021. The inclusion criteria were skin specimens with TEN confirmed in a pathology report and extent of lesions over 30% of the total body surface area (TBSA). From 35 patients with diagnosed in histopathology TEN 24 were enrolled into the study. The inclusion criteria were as follows: day of submission before 5th day from the onset of the symptoms, full protocol of plasmaphereses and IVIGs according to our scheme.

In our Centre we treat TEN with a combination of blood purification (plasmapheresis with fresh frozen plasma as a replacement fluid) and intravenous human immunoglobulins. Our protocol includes cycle of plasmapheresis with frozen fresh plasma twice daily for the first 2 days following admission, and once daily for the subsequent 5 to 7 days. IVIGs were administered after the first two sessions of plasmapheresis, for 4 to 7 days. The dosage was calculated according to body weight, at 0.4 to 0.5 g/kg per dose.

All patients were treated at the burn intensive care unit (ICU), according to the British Guidelines [21]. The Lund and Browder chart was used to evaluate the extent of the lesions.

We used non modified SCORTEN for evaluation of risk of death [18]. All included parameters were estimated at the admission to the ICU. Blood samples were taken at admission to the ICU, with SCORTEN scores estimated subsequentlyAll analysed parameters were taken at the admission to ICU. The analysed parameters included: age, sex, day of onset of symptoms, period between the onset of symptoms to admission to the ICU (in days), extend of lesions (in %of total body surface area TBSA), history of neoplasm (previous and current treatment), type of neoplasm, length of hospital stay (LOS). The criteria for hospital dismissal was full reepithelialisation. From the laboratory tests we collected data: haemoglobin (HGB), white blood cells count (WBC), creatinine, urea, glucose, HCO_3_, C- reactive protein (CRP), procalcitonin (PCT), total protein. From clinical parameters we evaluated heart rate (HR), systolic blood pressure, body temperature. We also analyzed previous symptomatic treatment, if any was administrated before admission to our ICU.

Data required for evaluation of SCORTEN were subjected to statistical analyses. SCORTEN is a sum of points in 7 categories, in each 1 point was added when: age was over 40, there was a history or a present malignancy, observed tachycardia was over 120, initial extent of skin lesions was over 10%, serum urea was over 28 mg/dl, serum glucose was over 252 mg/ml and bicarbonate levels were lower than 20 mEq/l. According to Bastuji-Garin a sum of 5 points and more equals mortality risk over 90% [17]. SCORTEN over 4 was recognised as a cut-off point for sensitivity and specificity evaluation.

The statistical analysis was performed with Statistica 13.1→ software (StatSoft, Poland). The descriptive statistics for the quantitative variables have been presented with the use of mean, standard deviation, median, upper and lower quartile, as well as minimum and maximum values. The qualitative variables have been presented by means of number and fraction values. The chi2 test was used to investigate the impact of qualitative variables. A 5% inference error margin was applied. Values of p < 0.05 were considered statistically significant. The Shapiro-Wilk test was used to examine variable distributions.

The principles outlined in the Declaration of Helsinki were respected. It was a retrospective study. The study protocol and individuals’ participation was approved by the Ethics Committee of the Independent Public District Hospital in Łęczna, nr 1/2022.

## Results

Patients’ characteristic is presented in Table 1. Beta lactams were the most frequent individual cause of TEN (3 cases). In 2 cases the causative agent was clindamycin, ibuprofen or carbamazepine. Most often there was one drug triggering blistering lesions (85.71%). The impact of the polypharmacy on death, as well as the type of causative drug, did not reach statistical significancy.

**Table 1:** Characteristic of the group

Number of patients	East Center of Burns Treatment and Reconstructive Surgery
	24
Female/Male (%)	13/11 (54.17/45.83)
Mean/median age	50.25/58.00 [range 14–82, SD 19.86]
Mean/median % TBSA	69.24/75.60 [range 30–96, SD 25.54]
History of neoplasm (%)	5 (20.83)
Mean/median day of subission	2.92/3.00 [range 1–4, SD 1.10]
Mean/median days of hospitalization	20.83/14.00 [range 4–55, SD 15.42]
Mean/median IVIG	181.25/200.00 [range 30–480, SD 103.61]
Mean/median	10.52/11.00 [range 2–21, SD 5.07]
**Number of plasmaphereses**	
Mean/median UREA	54.98/51.00 [range 17–133, SD 28.96]
Mean/median HCO3	27.32/25.70 [range 17.30–35.60, SD 5.51]
Mean/median GLUCOSE	129.46/124.50 [range 80–230, SD 28.84]
Mean/median HR	99.75/99.00 [range 65–155, SD 21.15]
Mortality	3 (12.50)
Mean/median SCORTEN	3.29/3.00 [range 1–7, SD 1.27]
Mean/median estimated death risk	41.93/35.30 [range 3.20–90, SD 23.60]

The mean age of all the patients was 50.2 [range 14–82, SD 19.86]. In our cohort age did not show statistically significant impact on mortality, p = 0.397, however survivors were younger than non-survivors (49 years vs. 55 years respectively). In our observation, the cut-off for death was 82 years.

The SCORTEN value at admission was 3.29 (mean), with an estimated mortality of 41,9%. The actual mortality rate observed was 12,5% (N = 3). Our treatment improved the survival, OR = 26,57, RR = 6,34, p = 0,022. None of the variables included as independent death risk factors in SCORTEN occurred to be a good prognostic agent on our cohort (Table 2).

**Table 2:** One-dimensional logistic analysis, parameters to calculate SCORTEN [18]

Variable	B	SE	Chi^2^ Wald	p	OR (95% CI)
AGE	-0,008	0,031	0,060	0,806	0,992 (0,933–1,055)
TBSA	0,019	0,029	0,435	0,228	1,019 (0,963–1,079)
CREATININ	3,881	2,221	3,052	0,081	48,476 (0,623–3770,945)
UREA	0,026	0,021	1,505	0,220	1,026 (0,985–1,070)
hco3	-0,184	0,146	1,582	0,208	0,832 (0,625–1,108)
GLUCOSE	0,005	0,020	0,064	0,800	1,005 (0,966–1,045)
HR	0,044	0,032	1,966	0,161	1,045 (0,983–1,112)

B - model parameters assessment SE - standard error Chi ^ 2 Walda - the value of the chi ^ 2 statistic checking the significance of parameters p - significance level for Wald’s test OR (95% CI) - odds ratio and 95% confidence interval

We also analysed other variables, describing laboratory results and clinical features at the admission, as well as day of submission, number of plasmaphereses and total dose of IVIGs (Table 3). None of the analysed parameters predicted gained statistical significance as a single death predictor. Also previous treatment and steroids before admission to ICU did not influence the final outcome (Table 4). A multivariate logistic analysis did not detect factors that would significantly predict death (Table 5). Clinical and laboratory results necessary for determining SCORTEN were analysed.

**Table 3:** One-dimensional logistic analysis

Variable	B	SE	Chi^2^ Wald	p	OR (95% CI)
Day of submission	0,083	0,583	0,020	0,886	1,087 (0,347–3,408)
IVIG dose	0,007	0,006	1,403	0,236	1,007 (0,996–1,018)
Nr of plasmaphereses	0,148	0,131	1,268	0,260	1,160 (0,896–1,500)
WBC	0,026	0,130	0,041	0,840	1,026 (0,796–1,323)
HGB	0,119	0,307	0,151	0,698	1,127 (0,617–2,056)
HCT	0,008	0,097	0,007	0,933	1,008 (0,833–1,220)
PLT	0,006	0,007	0,701	0,402	1,006 (0,992–1,020)
Albumin	-1,022	1,463	0,488	0,485	0,360 (0,020–6,335)
Protein	-0,427	1,123	0,144	0,704	0,653 (0,072–5,898)
CRP	-0,005	0,009	0,321	0,571	0,995 (0,978–1,013)
Temperature	-0,730	0,896	0,664	0,415	0,482 (0,083–2,789)
Ca	1,187	2,819	0,177	0,674	3,278 (0,013–823,050)
Amylase	0,001	0,003	0,189	0,664	1,001 (0,995–1,007)

B - model parameters assessment SE - standard error Chi ^ 2 Walda - the value of the chi ^ 2 statistic checking the significance of parameters p - significance level for Wald’s test OR (95% CI) - odds ratio and 95% confidence interval

**Table 4:** Logistic regression, influence of previous steroid submission on death

Variable	B	SE	Chi^2^ Wald	p	OR (95% CI)
Previous treatment	-0,981	1,302	0,568	0,451	0,375 (0,029–4,809)

**Table 5:** Multivariate logistic regression analysis, impact of variables on death

Variable	B	SE	Chi^2^ Wald	P	OR (95% CI)
AGE	-0,001	0,034	0,001	0,972	0,999 (0,935–1,067)
TBSA	0,019	0,030	0,409	0,522	1,019 (0,962–1,080)
UREA	-0,025	0,061	0,007	0,935	0,995 (0,883–1,121)
hco3	-0,405	0,296	1,870	0,171	0,667 (0,373–1,192)
GLUCOSE	0,022	0,040	0,318	0,573	1,023 (0,946–1,105)
HR	0,107	0,139	0,592	0,442	1,113 (0,848–1,460)

B - model parameters assessment SE - standard error Chi ^ 2 Walda - the value of the chi ^ 2 statistic checking the significance of parameters p - significance level for Wald’s test OR (95% CI) - odds ratio and 95% confidence interval

The sensitivity of the SCORTEN score for the entire cohort was 100%, with a specificity of 23,81%. In 85,71% of cases with low SCORTEN, death did not occur. High SCORTEN prognosed death accurately. Even though there was no statistically significant correlation between the age and the extent of lesions, we determined that the age of 82 and 96% of the TBSA were the cut-off points for the occurrence of death.

## Discussion

The SCORTEN scale consists of seven independent risk factors for death, including the extent of lesions at the onset [18, 19]. In our cohort the SCORTEN value at admission was 3.29, with an estimated mortality of 41,9%. The actual mortality rate was 12,5%. None of the variables included in SCORTEN reached statistical significance in our cohort. In our observation SCORTEN showed 100% sensitivity, and only 23,81% specificity. Independent risk factors did not reach statistical significance in our observation.

We use a combination of plasmaphereses and intravenous immunoglobulins. Our protocol improved the survival, OR = 26,57, RR = 6,34, p = 0,022. Differences between predicted and actual mortality, as well as a tendency for SCORTEN to overestimate the risk, were also observed by Imahara et al. [21].

The extent of lesions is one of the factors distinguishing TEN from SJS and SJS/TEN [2, 18], included in SCORTEN, where values in excess of 10% of the affected body surface area are an independent mortality risk factor [18]. Our cohort consisted of 24 patients with confirmed TEN, whereas in the Bastuji-Garin study TEN patients amounted to 41.8% of the group, therefore the study group was not homogenic. This might be one of the causes of the lower accuracy of the scale for the most severe cases. In a study by Zavala et al., TBSA and heart rate were not statistically significant predictors of death on days 1 and 3 [22].

Torres-Navaro et al. evaluated the current accuracy of SCORTEN in a meta-analysis of 64 papers [7]. SCORTEN scores lower than 3 overestimated mortality, whereas ones higher than 3, underestimated it. It is not only certain variables that are controversial, but the methodology used to determine SCORTEN is questionable as well [7]. The scale was created on the basis of a database from the 1970 and 1980 s, validated with data from the 1990s. Significant advances were made in supportive treatment and immunomodulatory therapy over the subsequent decades. With these developments and better sepsis control [8], different factors emerged. One of them is acute renal failure within the initial days of hospitalization [17], or a history of renal failure and dialysis prior to admission [11]. Renal insufficiency is not a novel risk factor, as it was described by Revuz in 1987 [23]. Bronchial necrosis and respiratory failure worsen the outcome as well [7]. Watanabe recognized late initiation of treatment at the specialty hospital as one of the mortality prognostic tools [24]. There is some evidence, however, that SCORTEN assessment can remain a valuable prognostic tool within the first 5 days after admission [21]. What is more, some authors use it later, to evaluate the efficacy of immunomodulatory treatment [22].

Age is another known death risk factor. In our cohort, survivors were younger than non-survivors (49 years vs. 55 years respectively). In our observation, the cut-off for death was 82 years, with the data not reaching statistical significance. In SCORTEN study, the mean age of the study group was 42.3 years, with the age cut-off established at over 40 years. In the ABCD-10 score, a prognostic scale created on the basis of the outcomes of 370 patients in a multi-institutional study conducted in the US, the cut-off age was 50 [24]. Thakur did not observe any impact of age on survival in SJS/TEN or TEN patients [25]. Watanabe determined age over 70 to be a death risk factor [24].

The accuracy of laboratory results evaluated in SCORTEN is also questionable [7, 26]. Hyperglycemia is one of the risk factors for sepsis and death in TEN. However, there are many variables that can influence serum glycemia [7]. In our ICU, all severe patients received parenteral nutrition and often required insulin intake. In such cases, the serum levels of glycemia, even within first 5 days of observation, are in our opinion an unreliable indicator. Our analysis did not prove serum glycemia to be a valuable death prediction factor in our cohort.

Blood urea nitrogen (BUN) is an independent mortality risk factor in critically ill patients [27]. BUN levels represent renal function but also neurohumoral activity in fluid homeostasis [27]. TEN pathogenesis is associated with dermal infiltration with dermoepidermal cytotoxic T-lymphocytes [1, 4]. Keratinocyte apoptosis and local inflammation [4] lead to subepidermal bullae, or epidermal necrolysis without destruction of dermal layers [2, 17]. The destruction of the external skin layer causes massive fluid loss and, as a consequence, hypovolemic and redistributive shock, similar to burn shock [28]. Non-inflammatory early acute renal failure is a common complication in severe burns affecting over 20% of the TBSA [29–31]. In TEN, however, early renal failure might be caused not only by loss of fluid, but also by circulating pro-inflammatory cytokines and complement [32]. Renal failure requiring renal replacement therapy before the onset of TEN symptoms is the most important factor burdening survival in the ABCD-10 score [11]. Renal function insufficiency at admission to the burn unit was corelated with a poorer outcome.

Low serum bicarbonate level is associated with higher mortality risk in chronic kidney disease. The NHANES survey, analysing cause-specific mortality, revealed that serum bicarbonate below 22 mEq/l was associated with a significantly worse survival. Low serum bicarbonate indicated a 46% higher risk of death among neoplastic patients [33]. Low bicarbonate levels in ICU patients are also a valuable indicator for predicting early mortality and risk of acute kidney injury [34]. Low bicarbonate levels showed to be the most important predicting factor for TEN patients [35]. Also in our cohort, low serum bicarbonate was associated with higher mortality.

The mortality rates among patients with SJS/TEN and a history of malignancy are worse [36]. According to SCORTEN, history of neoplasm is a mortality risk factor [14]. Wu et al. analysed malignancies in SJS/TEN patients. They discovered that phenytoin was more commonly used in the neoplastic group. The types of neoplasms that correlated with higher mortality risk were hepatocellular carcinoma and colorectal cancer [36]. However, it is not only the malignancy itself that should be seen as a risk factor, but also systemic treatment. The study showed that patients who underwent chemotherapy 3 months before the onset of SJS/TEN had worse survival [36]. Malignancies are often connected with malnutrition and pancytopenia, which are independent risk factors in SJS/TEN. Wound healing is also affected unfavourably [36]. We did not find a statistically significant relationship between history of neoplasm and higher mortality risk, but this might be due to limited data.

## Conclusion

SCORTEN showed low specificity and 100% sensitivity in our cohort. None of seven individual risk factors included in the score showed statistical significance in prognosing death in our TEN patients. The SCORTEN value at admission was 3.29, with an estimated mortality of 41,9%. The mortality rate within our cohort was 12,5%. Our data, as well as data from the available literature, suggest that the score predicting death among TEN patients should be revised, and new death risk factors should be included. Thorough analyses of the causes of death need to be performed in order to improve the available prognostic scores.

## Data Availability

Available in East Center of Burns Treatment and Reconstructive Surgery, Łęczna, Medical University of Lublin, or by the corresponding author dr.surowiecka@gmail.com.
